# Editorial: Women in virology: 2022

**DOI:** 10.3389/fmicb.2023.1244987

**Published:** 2023-07-24

**Authors:** Antoinette Cornelia van der Kuyl, Christine A. King, Ana Grande-Pérez

**Affiliations:** ^1^Medical Microbiology and Infection Prevention, University of Amsterdam, Amsterdam, Netherlands; ^2^Department of Microbiology and Immunology, Upstate Medical University, Syracuse, NY, United States; ^3^Instituto de Hortofruticultura Subtropical y Mediterránea “La Mayora” (IHSM-UMA-CSIC) Málaga, Málaga, Spain

**Keywords:** female scientists, STEM research, gender disparity, women, virology

Viruses have no gender, but virologists do. And, unfortunately, gender disparity in the STEM field continues, with, according to the UNESCO Institute for Statistics, only 30% of the world's researchers being women, and women being underrepresented at the highest levels of academia. As emphasized by UNESCO, science and gender equality are essential for a balanced future. To contribute to the laudable goal of creating a fairer world, and in celebration of International Women's Day 2022, Frontiers in Microbiology launched the Women in Virology 2022 Research Topic to highlight women's achievements in the field, and to act as a platform promoting the work of female scientists.

“*Women in virology: 2022*” was well received by the scientific community, with 13 manuscripts accepted for publication. In line with the broad call for submissions, the publications covered a variety of topics, describing both fundamental and clinical research on viruses infecting plants, oomycetes, and humans. An excellent overview by de la Higuera and Lázaro, is a valuable encounter with the world of viruses. The authors recap that viruses constitute the largest biomass on earth. With an estimated number of 10^31^ virus particles present, they greatly outnumber cellular organisms. Hundreds of millions of virions alone can be present in, for instance, a milliliter of seawater, and ≈20% of aquatic bacteria are lysed every day due to the actions of bacteriophages. Moreover, although it is impossible to draw a single phylogenetic tree of the Virosphere, the omnipresence of ancestral RNA-recognition motifs in viral replication proteins, which likely originate from the RNA world, viruses can be employed in studying the origin of life. The omnipresence and enormous diversity of viruses and their interaction with all organisms, further demonstrates their pronounced influence on the evolution of cellular life. For instance, viruses facilitate horizontal gene transfer in prokaryotes, while virus endogenization in eukaryotic genomes supports a role in evolutionary transitions. As viruses are found everywhere, are highly adaptable, easily dispersed by wind and water, and have a substantial influence on Earth's biochemical cycles and living organisms, the authors argue that, in the field of astrobiology, virus-like agents should be considered as potential carriers of life signatures, so-called “biosignatures,” when searching for extraterrestrial life.

Turning to viruses infecting humans, it is remarkable that nine out of ten contributors report on viruses that infect chronically or have the capacity to do so. Kaczorowska et al. report on mother-to-child transmission of the enigmatic anelloviruses (AVs), ubiquitous, persistent single-stranded DNA viruses with no known associated pathology. Sequencing the so-called anellome of five HIV-1 infected mother and child pairs suggested selected genotype transmission, as the majority of maternal lineages was not shared with the child. Some children harbored unique AV genotypes, likely acquired from other sources. In line with this finding, AV transmission of shared genotypes was not related to delivery or breastfeeding, as the HIV-positive mothers delivered by Caesarian section and did not breastfeed.

Five contributions investigated aspects of human papillomavirus (HPV) infection. In two papers, Yang et al., Yang et al. studied the epidemiology and risk factors associated with HPV infection and associated malignancies in women from Liaoning province in north-eastern China during 2018–2021, as in recent years, cervical cancer rates have been increasing in China, especially in younger women, and routine screening or vaccination is not standard practice. From a total of 16,589 participants, ±12.5% of women were found to be HPV+, with ±10% presenting abnormal cytology. For primary cervical cancer screening and ASCUS (atypical squamous cells of undetermined significance) triage, Li et al. compared the performance of a HPV DNA Chip test (Sample-to-Answer HPV Genotyping System from Beijing Bohui Innovation Biotechnology Co.) and an HPV PCR test (5 + 9 High Risk HPV Detection kit from Tellgen) on cervical samples from 7,241 women from rural areas in Shanxi, China during 2017–2018. Both tests performed better than standard liquid biopsy screening, while the HPV DNA Chip test (14.9% HPV+) outcompeted the HPV PCR test (21.1% HPV+) in the study. As the HPV situation in many parts of the world is critical, and prophylactic vaccination is not universal, Ren et al. performed a meta-analysis on the efficacy of prophylactic HPV vaccines among Asian females, and confirmed that these vaccines significantly reduce the incidence of persistent HPV infection, cytological abnormalities and cervical neoplasia in Asian populations. Unsurprisingly, the authors advocate raising awareness amongst women, and accelerated implementation of HPV vaccination. In a next study, Dai et al. found that stable expression of the HPV oncoproteins E6 and E7 in cell culture induces the differential expression of genes encoding chemokines and cell adhesion proteins. The observation may stimulate novel research into targeting E6E7 for HPV-related cancer treatment.

HBV is another example of a virus with an ability to persist and cause cancer in humans. Belaiba et al. investigated the genomic variation of hepatitis B virus (HBV) in five ETV (entecavir) non-responders in Tunisia. Putative drug-resistance mutations were present in the genotype D genomes, often predating treatment, as were a plethora of other mutations, some associated with disease progression and increased replication. Hepatitis C virus (HCV) is another potentially chronic hepatitis infection able to induce liver malignancy. García-Crespo et al. report a decrease in the effectiveness of antivirals when added, later rather than earlier, to an ongoing infection in cell culture. These results are important, as most patients begin treatment only when the infection has been established for some time, something which is not assessed in preclinical trials.

Human cytomegalovirus (HCMV) is another persistent virus infection. Herpesviruses are capable of alternating between lytic and latent cycles, whereby the induction of latency is an active process, in which major immediate early gene (IE) expression from the major immediate early promoter (MIEP) is suppressed, a process studied by Poole and Sinclair. They report that the cellular protein SERBP1 is upregulated during HCMV latency, is required for MIEP suppression and interacts with the transcriptional repressor CHD3 as well as with KAP1 to recruit SET1B, another repressor, to the viral genomic DNA, thus acting as a scaffold protein to maintain transcriptional silencing.

A non-persistent virus, the omicron-variant of SARS-CoV-2, was studied by Liu et al. who evaluated the presence of viral RNA in vaginal and anal swabs in 63 women with no, or only mild disease symptoms. All vaginal swabs were qRT-PCR negative for SARS-CoV-2 RNA. Four anal swabs were positive, one from a case with gastro-intestinal symptoms, suggesting that the omicron variant does not readily invade the gut.

A different section of the Virosphere was studied by Dye et al. who investigated the viral variation in cassava mosaic disease, an infection caused by a complex of single-stranded DNA viruses of the family *Geminiviridae*, genus *Begomovirus*. In the Kenyan Lake Victoria region, virus mixtures were found to be distinct from those in coastal samples. Experiments suggested that transmission dynamics of the viruses also differed, with African cassava mosaic virus (ACMV) being transmitted through vegetative propagation, while East African cassava mosaic virus (EACMV) relied on a whitefly vector. These findings could explain the divergent virus mixtures observed.

Raco et al. studied yet another realm of the virus world when searching for novel RNA viruses in the Vietnamese oomycete species, *Phytophthora castaneae*. Oomycetes are a group of fungus-like organisms with phylogenetic similarity to algae and diatoms, which include some of the most devastating plant pathogens. Five putative RNA virus genomes detected showed similarity to members of the order *Bunyavirales* and families *Endornaviridae, Megabirnaviridae, Narnaviridae, Totiviridae*, and the proposed family “Fusagraviridae”; a partial genome showed homology to the *Endornaviridae*. As initial virus identification was through small RNA sequencing, it was proposed that a RNAi antiviral defense mechanism had been targeting the viral RNA.

In conclusion, female virologists are as versatile as their subjects of study and work in all areas of the virosphere. Frontiers Research Topic “*Women in Virology*” helps showcasing their contributions to the field.

## Author contributions

AvdK drafted the manuscript. All three authors edited the Frontier's Research Topic “*Women in Virology 2022*”. All authors contributed to the article and approved the submitted version.

